# Confidence set of putative quantitative trait loci in whole genome scans with application to the Genetic Analysis Workshop 17 simulated data

**DOI:** 10.1186/1753-6561-5-S9-S58

**Published:** 2011-11-29

**Authors:** Charalampos Papachristou

**Affiliations:** 1 Department of Mathematics, Physics, and Statistics, University of the Sciences, 600 S. 43rd Street, Philadelphia, PA 19104, USA

## Abstract

As genetic maps become more highly dense, the ability to sufficiently localize putative disease loci becomes an achievable goal. This has prompted an increased interest in methods for constructing confidence intervals for the location of variants that contribute to a trait. Such intervals are important because, by reducing the number of candidate loci, they can help in the design of cost-effective and time-efficient follow-up studies. We introduce a new approach that can be used in whole-genome scans to obtain a confidence set of loci that contribute at least a predetermined percentage *h* to the overall genetic variation of a quantitative phenotype. The method is developed in the framework of generalized linear mixed models and can accommodate families of arbitrary size and structure. We apply our method to the Genetic Analysis Workshop 17 simulated data where we scan chromosomes 6, 15, 20, 21, and 22 to uncover loci regulating the simulated phenotype Q2. For the analyses we had prior knowledge of the simulation model used to generate the phenotype.

## Background

Technological advances have provided us with highly dense genetic maps of single-nucleotide polymorphisms (SNPs) covering the whole genome. Based on these maps and using a variety of statistical methods, scientists nowadays routinely scan the human genome in search of loci that either contribute to the variability of quantitative phenotypes or predispose individuals to develop binary traits. Currently used approaches have been particularly successful in identifying mutations in genes that cause relatively rare hereditary diseases. However, the search for susceptibility genes for common diseases has proved to be more challenging. It is widely accepted that such complex traits are influenced by many loci, each having only a small contribution to the phenotype. Naturally, the detection of these genes is difficult. Furthermore, investigators must adjust for multiplicity of the large number of markers simultaneously tested. This is not a trivial task considering the complex dependencies in genetic data. As such, most genome-wide association studies suffer from low power and often point to large genomic regions. Identifying the genes in these regions and locating those associated with the trait of interest can be both time-consuming and expensive. Hence there is an increased interest in methods that can significantly reduce the number of candidate genes identified but that have sufficient power, in the preliminary scan, to aid in the design of more cost-effective and time-efficient follow-up studies.

We propose a new confidence set inference (CSI) method that is motivated by the fact that, because the new genetic maps are highly dense, it is expected that many SNPs will not only reside within disease genes but also may be the causative variants themselves. Our approach can be used in preliminary genome association studies to obtain a confidence set (CS) of quantitative trait loci (QTLs) contributing *at least* a predetermined percentage *h* to the overall genetic variation of a quantitative phenotype. The method is developed in the framework of linear mixed models (LMMs) and can accommodate families of arbitrary size and structure. Furthermore, the approach provides a flexible framework that allows one to search for loci that have at least a specific level of contribution to the quantitative trait. As such, it gives us the ability to set the bar higher or lower depending on the amount of data available.

## Methods

### Hypotheses and test statistic

In traditional family-based association mapping the null hypothesis is that there is no association, whereas the alternative is that there is association. In our formulation we are actually reversing these two hypotheses. For each SNP on the map, our null hypothesis is that the locus is a QTL contributing at least a certain percentage of the total genetic variance  of the quantitative phenotype, whereas the alternative is that the locus contributes to the trait less than the prespecified level (or nothing at all). More specifically, we assume that there is a dense SNP map consisting of *S* markers that potentially harbor some QTLs which contribute to the phenotype of interest. Then, for each SNP *s* (*s* = 1, …, *S*) we test the following hypotheses:(1)

where  is the genetic variance attributed to SNP *s* and *h* is a number between 0 and 1 and is chosen in advance. It follows that the set of markers for which the null hypothesis in Eq. (1) is not rejected at level *α* constitutes a (1 − *α*) × 100% confidence set of loci contributing at least *h* × 100% to the total genetic variance of the phenotype.

Note that because of the reversal of the traditional null and alternative hypotheses, the type I error and power of our method are also the reversals of the traditional ones. To avoid any confusion, in the rest of this paper we use the term *true positive* to refer to any SNP that is included in the confidence set and is a trait-regulating locus. Similarly, we use the term *false positive* to denote any SNP that is included in the confidence set and does not contribute to the trait.

We briefly describe how we test the hypotheses in Eq. (1). Consider a pedigree of arbitrary structure consisting of *n* members. Let *y* = (*y*_1_, …, *y_n_*) be the vector of family phenotypic values of the trait of interest. We assume that for each person *i* the value of his/her phenotype is governed by a major locus *τ*. Furthermore, the trait value is also influenced (potentially) by some known covariates *X_i_* (e.g., age and sex), which includes a constant of 1 to signify the overall effect. In addition, there is some environmental effect or residual, denoted *e_i_*. Finally, we assume that the phenotype is affected by a number of other loci whose collective (random) polygenic effect on the trait is denoted *u_i_*. Now, assuming additivity across all effects, the overall phenotypic value of a person is:(2)

where *β* is a vector of unknown coefficients, *γ* is the coefficient of the effect of the major locus, and *z_τ_* is the number of copies of the disease allele at the major trait locus carried by the individual. We assume that the vector of random polygenic effects *u* = (*u*_1_, …, *u_n_*) follows a multivariate normal distribution with mean 0 and covariance matrix *Ω*, that the *e_i_* are independent and identically distributed from a normal distribution, also with mean 0 and variance , and that the two random effects are independent of each other and the covariates. Under these assumptions, the joint distribution of the *y_i_* is simply a multivariate normal distribution with mean equal to *βX* + *γz_τ_* and variance covariance matrix:(3)

where *X^T^* = (*X*_1_, …, *X_n_*) and *I* is the *n* × *n* identity matrix.

If the dominance genetic variance is negligible and there is minimal or no inbreeding in the family, then the matrix *Ω* is approximately equal to:(4)

where *Φ* is a known matrix whose elements are the kinship coefficients between the family members and  is the additive polygenic variance [1,2]. If we further assume that the major trait locus *τ* is diallelic with minor allele frequency *p_τ_*, then we can show that the coefficient *γ* in Eq. (2) must satisfy:(5)

where  is the additive genetic variance due to the major locus *τ* and *p_τ_* is the frequency of the disease allele. Using this fact, we can see that the hypotheses in Eq. (1) are equivalent to:(6)

where  is the total additive genetic variance of the quantitative trait, *p_s_* is the frequency of the minor allele of the SNP *s*, and *γ_s_* is the same coefficient as in Eq. (2) when *s* is the major trait locus. The statistic for testing the above hypotheses is given by:(7)

where  and  are the maximum-likelihood estimate of *γ_s_* and its standard deviation, respectively, obtained by maximizing the multivariate normal likelihood that corresponds to the LMM in Eq. (2). It is easily seen that the (asymptotic) distribution of the test statistic is a standard normal distribution. Thus we can easily find an appropriate threshold for testing the hypotheses in Eq. (6) at any significance level *α*.

Finally, the last piece of information we need to perform the hypothesis test is the value of the overall total additive genetic variance of the phenotype . This can be readily estimated from the data themselves, usually with good accuracy, by maximizing a similar likelihood as in Eq. (2) but without any major gene effects [1,2].

The construction of the confidence set of SNPs that contribute at least a specific proportion *h* to the total genetic variance of a quantitative trait is achieved as follows. First, we obtain an estimate  of the overall genetic variance by fitting the model in Eq. (2) without any effects of major genes, that is, without the parameter *γ*. Next, for each SNP *s*, we obtain the maximum-likelihood estimate of *γ_s_* and its standard deviation,  and , respectively, by maximizing the likelihood in Eq. (2). Then, for each SNP *s*, we use *h*, , , and  to compute the test statistic *t_s_*. Finally, a (1 − *α*) × 100% confidence set of loci contributing at least *h* × 100% to the total genetic variance of the phenotype confidence set is formed by aggregating all those SNPs for which |*t_s_*| >*z_a_*, where *z_α_* is the upper *α*th percentile of the standard normal.

## Results

We used the family simulated data sets from Genetic Analysis Workshop 17 (GAW17) [3] in an attempt to localize loci that are related to the simulated quantitative phenotype Q2. Under the simulation model, 72 SNPs contribute to the levels of the quantitative phenotype. Because of time limits, we chose to analyze chromosomes 6, 15, 20, 21, and 22 for all 200 replicates provided in the simulated data. We selected chromosome 6 because it houses nine SNPs that contribute to the phenotype, and thus it can help us gauge the ability of our method to identify loci contributing to the trait. Chromosomes 15, 20, 21, and 22 harbor no loci regulating the trait, and they are used to study the false discovery rate (FDR) of the method. We preprocessed the data and excluded from the analysis SNPs with a minor allele count less than 28 copies in the entire data set (roughly minor allele frequencies [MAFs] of 4%, 2%, 1.5%, and 1% for the single, double, triple, and quadruple data set, respectively) in order to avoid potential problems with unstable estimates of the model parameters in Eq. (2) for SNPs with rare minor alleles. Because we analyzed only the simulated data, we did not consider any filtering criterion that was based on the genotyping rate, calling rate, or Hardy-Weinberg equilibrium. After preprocessing the data, we were left with 351 SNPs on chromosome 6, 189 SNPs on chromosome 15, 137 SNPs on chromosome 20, 51 SNPs on chromosome 21, and 99 SNPs on chromosome 22. Furthermore, only three causative SNPs (C6S5380 on the *VNN1* gene and C6S5426 and C6S5441 on the *VNN3* gene) remained on the reduced map out of the nine SNPs that reside on chromosome 6.

Each of the 200 replicates of the family data consists of genotypes and phenotypes on 697 individuals forming 8 extended pedigrees with the number of members ranging from 73 to 128. This sample size may not be sufficient to provide enough power for our method to significantly localize the loci that contribute to the trait. Thus we decided to artificially increase the sample size by combining data from 2, 3, or 4 consecutive replicates to create 100, 66, and 50 new replicates of samples with 1,394, 2,091, and 2,788 individuals, respectively. Note that the replicates are not completely independent because they share the same genotypes. This could potentially affect the performance of the method. However, the independence of the phenotypes should help to moderate the effect of the common genotypes.

Using our method and these new data sets as well as the original 200 replicates, we constructed 95% confidence sets for loci that contribute a specific percentage *h* to the overall additive genetic variance. The model we used to analyze the data assumed only the additive genetic variance component and only one covariate, the overall mean level of Q2. Even though additional covariates, such as age, sex, and smoking status, were available, a small preliminary analysis on several replicates showed that none of the covariates were statistically significant. Hence we opted to avoid including them in this particular study to ease the computational burden. Finally, even though the family sizes were significantly large, 75 to 128 individuals per family, our method was able to handle all the pedigrees as a whole without splitting them into smaller units.

In Table 1 we summarize the results from the analyses of all replicates for the four different sample sizes. For each data set and each threshold *h*, we report the true discovery rate (TDR) (the proportion of replicates that yield 95% confidence sets that include at least one SNP that contributes to the trait) and the FDR (the proportion of replicates for which the resulting 95% confidence set includes only nonfunctional SNPs). The last four columns of the table report the mean and standard deviation of the number of causative (true-positive) and noncausative (false-positives) SNPs that were included in the 95% confidence sets and that were based on all those replicates that yielded nonempty confidence sets. For each data set, we present the results for two different thresholds of *h*. The first threshold corresponds to the smallest level for which the method yielded an FDR as close to 0.05 as possible, without exceeding it; the second threshold is the minimum threshold for which the method yielded zero FDR. For instance, for the triple data set (2,091 individuals) we chose the thresholds 0.058 and 0.086 because 0.058 resulted in confidence sets that had no functional SNPs in 3 out of the 66 replicates (FDR of 0.045) and because 0.086 resulted in confidence sets that had only causative loci.

**Table 1:** Analysis results for the 95% confidence sets of loci contributing to the quantitative phenotype Q2

				Causative SNPs^e^		Noncausative SNPs^f^	
				
Data set^a^	*h*^b^	TDR^c^	FDR^d^	Mean	SD	Mean	SD
Single (697)	0.176	0.025 (0.020)	0.050	0.33	0.13	1.40	0.49
	0.250	0.000 (0.000)	0.000	0.00	0.00	0.00	0.00
Double (1,394)	0.092	0.180 (0.160)	0.050	0.83	0.10	0.30	0.15
	0.120	0.040 (0.040)	0.000	1.00	0.00	0.00	0.00
Triple (2,091)	0.058	0.697 (0.515)	0.045	1.18	0.08	0.41	0.14
	0.086	0.076 (0.076)	0.000	1.00	0.00	0.00	0.00
Quadruple (2,788)	0.045	0.860 (0.640)	0.040	1.36	0.09	0.44	0.16
	0.060	0.540 (0.520)	0.000	1.07	0.05	0.04	0.04

The results are based on 200 single, 100 double, 66 triple, and 50 quadruple replicates.

^a^ Number of original replicates combined to form the new data set to be analyzed. The number in parentheses corresponds to the number of individuals in the sample.

^b^ Threshold for contribution of a putative locus to the total additive genetic variance.

^c^ Proportion of replicates for which the resulting 95% confidence set includes at least one trait locus. Numbers in parentheses give the proportion of replicates for which the resulting confidence sets include only trait loci.

^d^ Proportion of replicates for which the resulting 95% confidence set includes only noncausative SNPs.

^e^ Observed mean and standard deviation (SD) of the number of causative SNPs included in the resulting 95% confidence sets per replicate.

^f^ Observed mean and standard deviation (SD) of the number of non-causative SNPs included in the resulting 95% confidence sets per replicate.

As expected, a sample size of 697 (single) or 1,394 (double) was not enough for our method to be able to distinguish between causative and noncausative SNPs. To bring the FDR to zero levels, we had to set *h* to 0.25 for the single replicate and 0.12 for the double replicate. These high thresholds, though, caused the TDR to become almost zero.

However, when the sample size was tripled to 2,091 people, the advantages of our method started to unfold. An *h* of 0.058 was sufficient to hold the FDR to acceptable levels (less than 5%). At this threshold level, we were able to identify at least one QTL in chromosome 6 in 46 out of the 66 replicates, a TDR of almost 70%. In fact, in 34 of the replicates (51.5%) the 95% confidence set included only causative SNPs. On average, we were able to identify 1.18 causative SNPs, and the confidence set included on average 0.41 noncausative SNP, thereby demonstrating the ability of the method to target only loci that contribute to the trait. When we increased the threshold *h* to 0.076, the FDR of the method dropped to zero, but the TDR was also significantly reduced to roughly 8% (5 replicates out of 66). Not surprisingly, all five of these replicates resulted in confidence sets that included only causative SNPs.

A further increase in the sample size to 2,788 individuals (quadruple set) resulted in a significant increase in the observed TDR of the method. To reduce the FDR to close to 5%, the threshold for *h* needed to be 0.045, and to reduce FDR to 0, *h* had to be 0.060, which resulted in TDRs of 86% (43/50) and 54% (27/50), respectively. Again, most of the replicates (32 for the 0.045 threshold and 26 for the 0.060 threshold) yielded 95% confidence sets that included only causative SNPs.

Finally, the confidence sets included on average less than 0.5 nonfunctional SNP. Note that a large number of these false-positive SNPs came from chromosome 6, especially for the smaller sample sizes (single and double sets). A closer investigation revealed that these were usually SNPs that were in high linkage disequilibrium with the causative SNPs residing on chromosome 6. Nevertheless, as the sample size increased, our method was able to distinguish the causative SNPs from those in high linkage disequilibrium with them, thereby demonstrating a significant ability to localize causative loci. For example, for the quadruple data set with a threshold of 0.06, the CSI method identified causative SNPs in only 26 out of the 50 replicates (52%).

## Discussion and conclusions

We have presented a CSI method for obtaining a set of SNPs that contribute at least a prespecified percentage *h* to the total additive variance of a quantitative trait. Our method provides a flexible tool that can be used in preliminary whole-genome association scans to significantly reduce the number of candidate genes that need to be followed up, thereby making subsequent studies more time- and cost-efficient. The key idea of our method lies in the reversal of the traditional null and alternative hypotheses of association versus nonassociation. The main advantage of reversing the traditional hypotheses is that it enables researchers to target genes with a specific contribution to the genetic variance of a trait and at the same time to choose the confidence level needed to identify at least one such gene, if it exists. Having the ability to control this confidence level is particularly useful in preliminary studies. Such studies aim to reduce the number of SNPs that will be carried over to the second stage of the analysis and strive to ensure, with high confidence, that enough causative loci will also be carried over. Thus our method is particularly suitable for preliminary scans involving a large number of SNPs that densely cover the entire genome.

Further advantages of our method include the ability to handle families of arbitrary size and structure and the ability to incorporate into the analysis information on pertinent variables that may have an effect on the quantitative phenotype. Finally, our method is expected to be robust with respect to population stratification because it uses data from extended families, and family-based association tests tend to be robust with respect to population stratification [4]. Application of our method to the GAW17 simulated data demonstrates that our method has the ability to significantly localize putative quantitative trait loci with high accuracy, down to within two SNPs, provided that there is a sufficient amount of data (at least 2,000 individuals), while maintaining a low false-positive rate.

Implementation of our method requires knowledge of the overall additive genetic variance of the trait and the allele frequencies of the SNPs. This information can be readily obtained from the data themselves, usually with high accuracy, depending on the sample size. An important tuning parameter in the implementation of our method is the choice of the threshold *h* of the contribution of the putative trait loci to the overall additive genetic variance. This level *h* needs to be selected in advance, but its value should depend on the amount of data available for the study. Small sample sizes will require higher values of *h* to warrant a low FDR, whereas larger sample sizes, in practice more than 2,000 people, may allow for the identification of loci with a contribution as low as 5% to the total additive genetic variance. In principle, one could use a bootstrap approach to obtain thresholds tailored to the particular data used. However, for whole-genome scans involving a large number of individuals, the computational burden associated with bootstrap methods may render such an approach infeasible. In such a case, one may limit the number of SNPs to be followed up by adopting the following practical approach: Using our method, researchers can obtain 95% upper confidence limits for the contribution of each SNP to the total additive variance of the trait. Then, they can rank the loci according to these upper bounds and select the top loci for further study.

The current formulation assumes additivity across the different loci that regulate the trait and within the locus of interest. Even though additive genetic models have been considered a good approximation to more complicated inheritance modes, our method could be extended to accommodate dominant or recessive models. Finally, in this study we focused on loci that had relatively common functional variants (at least 28 copies of the minor allele). Considering more rare variants significantly increases the FDR of the method. Probably, the insufficient number of people with rare genotypes in the samples led to misestimation of the model parameters, which in turn increased the FDR. Perhaps, for more rare variants there is a need for larger sizes to ensure that enough individuals with the rare allele are included.

## Competing interests

The author declares no competing interests.

## Author’s contributions

The author developed the methodology presented in the contribution, performed the analyses, and drafted the manuscript. The Author read and approved the final manuscript.
